# Implementation and scaling-up of an effective mHealth intervention to increase adherence to triage of HPV-positive women (ATICA study): perceptions of health decision-makers and health-care providers

**DOI:** 10.1186/s12913-023-09022-5

**Published:** 2023-01-18

**Authors:** Cecilia Straw, Victoria Sanchez-Antelo, Racquel Kohler, Melisa Paolino, Kasisomayajula Viswanath, Silvina Arrossi

**Affiliations:** 1 Faculty of Social Sciences, University of Buenos Aires, Buenos Aires, Argentina; 2 Centre for the Study of State and Society, Buenos Aires, Argentina; 3 Centre for the Study of State and Society, National Council for Scientific and Technical Research, Buenos Aires, Argentina; 4 Cancer Health Equity, Cancer Institute of New Jersey, Rutgers - the State University of New Jersey, New Brunswick, USA; 5 Department of Social and Behavioral Sciences, Harvard T.H. Chan School of Public Health, Harvard University, Boston, USA

**Keywords:** mHealth, Self-collection HPV, Adoption, Scaling-up, Implementation

## Abstract

**Background:**

The ATICA study was a Hybrid I type randomized effectiveness-implementation trial that demonstrated effectiveness of a multicomponent mHealth intervention (Up to four SMS messages sent to HPV-positive women, and one SMS message to CHWs to prompt a visit of women with no triage Pap 60 days after a positive-test), to increase adherence to triage of HPV positive women (ATICA Study). We report data on perceptions of health decision-makers and health-care providers regarding the intervention implementation and scaling-up.

**Methods:**

A qualitative study was carried out based on individual, semi-structured interviews with health decision-makers (*n* = 10) and health-care providers (*n* = 10). The themes explored were selected and analyzed using domains and constructs of the Consolidated Framework for Implementation Research (CFIR) and the maintenance dimension of the Reach Effectiveness Adoption Implementation Maintenance (RE-AIM) framework.

**Results:**

Both health-care providers and decision-makers had a positive assessment of the intervention through most included constructs: knowledge of the intervention, intervention source, design quality, adaptability, compatibility, access to knowledge and information, relative advantage, women’s needs, and relative priority. However, some potential barriers were also identified including: complexity, leadership engagement, external policies, economic cost, women needs and maintenance. Stakeholders conditioned the strategy’s sustainability to the political commitment of national and provincial health authorities to prioritize cervical cancer prevention, and to the establishment of the ATICA strategy as a programmatic line of work by health authorities. They also highlighted the need to ensure, above all, that there was staff to take Pap tests and carry out the HPV-lab work, and to guarantee a constant provision of HPV-tests.

**Conclusion:**

Health decision-makers and health-care providers had a positive perception regarding implementation of the multicomponent mHealth intervention designed to increase adherence to triage among women with HPV self-collected tests. This increases the potential for a successful scaling-up of the intervention, with great implications not only for Argentina but also for middle and low-income countries considering using mHealth interventions to enhance the cervical screening/follow-up/treatment process.

**Supplementary Information:**

The online version contains supplementary material available at 10.1186/s12913-023-09022-5.

## Introduction

Cervical cancer (CC) is a preventable disease with existing evidence and technologies. For this reason, the World Health Organization (WHO) has launched a worldwide initiative to eliminate CC through vaccination, screening and treatment [1]. In Argentina, every year 4600 new cases of CC are diagnosed, and some 2600 women die due to the disease [2].

Human papillomavirus (HPV) DNA testing is a highly effective screening method to prevent CC [3] that allows women to self-collect samples. HPV self-collection has been shown to increase screening uptake among socially vulnerable women [4], especially when offered by community health workers (CHWs) during home visits [5]. In an HPV self-collection program, the triage of HPV-positive women is a key step in identifying who will need to continue with diagnosis and treatment.

Nevertheless, the low adherence to triage and treatment is a longstanding problem for CC programs in low- and middle-income countries (LMIC) [6], especially among women with limited access to health care [7]. In Argentina, where HPV self-collection was introduced as a programmatic strategy in 2014, adherence to triage continues to be a challenge [7, 8]; only around 25% of women with HPV-positive self-collected tests successfully triage within 120 days after screening positive [8]. One key issue is the delivery of test results and referral of HPV-positive women for triage [9]. In the case of self-collection, receiving results is challenging, as women collect their samples offered by CHWs during home visits. Given that CHWs are a scarce human resource responsible for the provision of a variety of health services, it is often not viable for them to re-visit all HPV + women (around 13% of all tested women) to deliver results and refer them to health centers for triage [8]. Therefore, effective interventions aimed at improving triage adherence that do not require the intensive use of human resources in health are needed.

Mobile phone text messages (SMS messages) are useful to remind patients about medication adherence, such as antiretroviral therapy and asthma treatment [10–12], and in reducing non-attendance rates to preventive health care centers in many LMIC [11, 13–15]. Similarly, mHealth tools targeted at providers, such as SMS reminders for CHWs, have been shown to improve the quality of service provided to the population, mostly through decision support, alert and reminder tools [16–19]. SMS messages have advantages over other reminder systems, including that they can be sent to patients simultaneously and require less staff [10, 13, 20]. SMS reminders are easy to use and useful for patients [10, 20, 21]. The ATICA Study (Application of Communication and Information Technologies to Self-Collection, for its initials in Spanish) is a hybrid type I cluster randomized effectiveness-implementation trial (C-RCT) to evaluate the effectiveness of a multi-component mHealth intervention (ATICA strategy) to increase adherence to triage by women with a positive self-collected test [22]. ATICA C-RCT results showed that SMS sent to HPV-positive women combined with e-mail/SMS sent to CHWs to visit women without Pap triage after 60 days effectively increased triage [23]. For this reason, scaling-up of the ATICA strategy into routine CC screening programs using self-collected tests could have great impact in CC prevention programs by facilitating the continuity of care for HPV-positive women.

Evidence indicates that in low-middle income countries, adoption of innovations is unreliable and slow, and there is a dearth of evidence about what factors influence the delivery of evidence-based interventions at scale [24]. Incorporation of an evidence-based innovation as ATICA strategy into the health system depends on the potential users’ intention to utilize the innovation, the set of practices and processes that make up the implementation, and, in particular, is highly influenced by the perception of the actors involved in the intervention’s implementation and use [25]. In ATICA study, both acceptability of the intervention by HPV-tested women, and its adoption by CHWs were high [23]. However, incorporation of the multicomponent intervention as a routine programmatic public health policy will also depend on health authorities’ and health care providers’ (HCP) acceptability and perceptions about barriers and facilitators of the intervention implementation. However, to our knowledge, very few evidence exists regarding these key stakeholders’ perspectives about using mHealth interventions to increase adherence to triage of women with HPV self-collected tests. In this paper, we present an evaluation of health decision-makers’ and health-care providers’ (HCPs) perceptions about implementation of the ATICA strategy. This qualitative analysis allows for a better understanding of key factors for scaling up ATICA strategy and builds on the effectiveness data from the C-RCT [23].

## Methods

### Study setting

The province of Jujuy is located in northwest Argentina. It has approximately 673,000 inhabitants, of which 51% are women, 87% live in urban areas, 32% are poor, and approximately 45% have public health insurance [26]. Mobile phone penetration in urban areas was 86% in 2019 [27].

The public health system includes a primary hospital and 270 primary health care (PHC) centers. The PHC system employs about 700 full-time CHWs that visit approximately 110,000 homes twice a year to carry out health-related tasks such as vaccination and child-maternal health promotion [22].

Jujuy CC prevention, screening and treatment program has been extensively described elsewhere [28]. Briefly, since 2012 HPV-testing is the primary screening method for women 30 years and older who receive care in the public health sector. In 2014, self-collection was introduced as a screening strategy for women with public health insurance and not tested in the previous 5 years [22]. CHWs offer self-collection during home visits. Women who perform self-collection are told to visit a health center within 30 days to receive the results, and if results are positive, to carry out cytology-based triage in the health center [22]. All testing/diagnosis/treatment procedures performed in the public health system are registered in the national screening information system (SITAM, for its initials in Spanish) to generate a diagnosis form, which is instantly available online to all providers at public health establishments.

The ATICA study was rooted in programmatic, real-world conditions described above; its methods have been extensively described elsewhere [22]. Briefly, ATICA was an effectiveness-implementation hybrid type I trial, combining a C-RCT to evaluate the effectiveness of a multi-component mHealth intervention with surveys of CHWs and HPV-positive women, and semi-structured interviews with local health authorities and health providers to evaluate the intervention implementation. CHWs (clusters) belonging to PHC were randomly allocated to a multi-component intervention (up to four SMS messages sent to HPV-positive women, and one SMS message to CHWs to prompt a visit of women with no triage Pap 60 days after a positive-test), or control group (Usual care). The primary effectiveness outcome was percentage of HPV-positive women with triage 120 days after the HPV-test result. 132 CHWs in the intervention group invited 3272 women to participate. Once women had performed HPV self-collection, CHWs explained the sequence and content of SMS messages, and that they might come back for a personal visit. Women recruitment took place between December 4, 2018 and July 31, 2019. A total of 445 women in the intervention group had an HPV-positive result (13·7%) and were sent the SMS [23].

In addition, we evaluated implementation of the intervention using the Reach, Effectiveness, Adoption, Implementation, Maintenance (RE-AIM) framework [23]. Data sources and results of RE-AIM based analysis have been published elsewhere. Surveys were carried out among HPV-positive women and CHWs from the intervention group to evaluate their acceptability and perceptions regarding the intervention. Results showed that 97.2% of women accepted the ATICA strategy. In addition, adoption by CHWs was high, as 86.9% of CHWs agreed with programmatic incorporation of the mHealth intervention and 90.8% adopted the intervention [23].

### Conceptual frameworks

In this paper, we present results from the semi-structured interviews with HCP and decision makers carried out to gather information about their perceptions regarding the intervention implementation The themes explored were selected and analyzed using the domains and constructs of the Consolidated Framework for Implementation Research (CFIR) [25], and the maintenance dimension of the RE-AIM [29], as presented in Table 1. The CFIR provides a menu of theoretical and multidisciplinary constructs that allow for clear and consistent articulation of factors that may potentially affect the results of implementation [25, 30]. Since it was developed, their constructs have been expanded and are currently being used for designing, implementing, and evaluating various healthcare strategies in different contexts and countries. The RE-AIM framework is aimed at expanding assessment of interventions beyond efficacy to multiple criteria that may better identify the translatability and public health impact of health promotion interventions [29]. In the last years RE-AIM has been largely used to increase visibility of the context in which results referred to main outcomes of health interventions are obtained. Maintenance refers to the extent to which a program or policy becomes institutionalized or part of the routine organization practices and policies [31]. Thus, in this study the maintenance dimension of the RE-AIM was included to provide evidence about HCP and decision-makers’ perceptions regarding the potential sustainability of the intervention.

**Table 1:** CFIR and RE-AIM Domains, constructs and definitions

CFIR Domains	Construct		Definition
I. Intervention characteristics	Intervention Source		The legitimacy of ATICA as an intervention external to the institution to which the HCPs and decision-makers belong. Legitimacy of the institutions to lead the implementation and adoption of the intervention.
	Relative advantage		The advantage of implementing ATICA versus an alternative solution and/or current usual practices.
	Adaptability		The degree to which the ATICA components can be adapted or refined to meet local needs.
	Complexity		The perceived difficulty of the intervention.
	Design quality		Perceived excellence in how the intervention was bundled, presented, and assembled.
	Cost and resources		The costs of the intervention and costs associated with its implementation, including investment, supply and opportunity costs.
II. Outer Setting	Patient needs and resources		The perception of the degree to which ATICA responds to the patients’ needs.
	External policies and incentives		Strategies to promote ATICA through policies and regulations.
III. Inner Setting	Access to knowledge and information		Ease of access to information and knowledge about the intervention and how to incorporate it into work tasks.
	Implementation climate	Compatibility	The compatibility of ATICA with the norms, values, and existing workflows and systems.
		Relative priority	The individuals’ shared perception of the importance of the ATICA implementation within the institution.
	Readiness for Implementation	Leadership engagement	Commitment, involvement, and accountability of health authorities with regard to the ATICA implementation.
IV. Characteristics of Individuals	Knowledge and beliefs about the intervention.		Knowledge and attitudes regarding the ATICA strategy.
**RE-AIM Dimension**			
Maintenance			The extent to which a program or policy becomes institutionalized or part of the routine organization practices and policies.

Own elaboration based on an adaptation of Damschroder [25] and Glasgow [29]

### Target population and sampling

The sampling was purposive. Sample size (N = 20) was based on relevance and theoretical saturation [32]. An important characteristic of the sample was the role played by the interviewees in CC cancer prevention policies and activities at provincial level. We recruited health decision-makers as to include coordinators of PHC, HPV laboratory, and Women Health Department, the director of the provincial Program on Cervical Cancer Prevention, heads of Health Secretariats, and heads of gynecology services. In addition, we interviewed HCPs belonging to the public health system, and working at PHC where they routinely performed triage Paps. To avoid potential bias in responses due to a double role, those decision-makers who performed healthcare functions at PHC level were excluded; as well as HCPs who were involved in managing cancer control and prevention policies.

### Instrument for information collection

Individual semi-structured interviews were conducted using a guide based on included constructs of CFIR and RE-AIM (Table 1). Questions related to the external policies and costs constructs were only asked to decision-makers.

### Data collection

Interviews were conducted between December 2020-February 2021 by a female social science researcher. She invited stakeholders through e-mail/WhatsApp to participate in an online interview through a virtual platform (Zoom). Interviews lasted on average one hour, permission was obtained from participants to audio-record them. Interviewees were shown three cards illustrating the ATICA multi-component mHealth intervention and content of the SMS [33].

### Analyses

The collected data were analyzed thematically [34]. Once the interview transcriptions were completed, all interviews were entered into the software ATLAS.ti (version 7.5.4; ATLAS.ti *Scientific Software Development GmbH*, Berlin). The coding and analysis were done in accordance with the domains, constructs and dimensions presented in Table 1. In the analysis the perspectives of health decision-makers and HCPs were compared; points of agreement and disagreement were highlighted.

The analysis was carried out by two researchers; the results were discussed in joint sessions with the research team, and disagreements were resolved through discussions.

The details of the methods and results of the semi-structured interviews are presented following the Consolidated Criteria for Reporting Qualitative Studies [35].

### Ethical aspects and participant’s consent

The research study was performed in accordance with the Declaration of Helsinki. ATICA study’s protocol including research on health decision-makers and HCPs perspectives was approved by CEMIC Institutional Review Board, the Ethics Research Committee of Jujuy Ministry of Health, The Institutional Review Board of Harvard T.H. Chan School of Public Health, and Deakin University Human Research Ethics Committee. Before the interview, the study objectives were explained to each participant who then signed an informed consent form. The confidentiality and anonymity of the participants was guaranteed.

## Results

Ten health decision-makers and 10 HCPs were interviewed. The average amount of time that health decision-makers had occupied their roles was 11 years (range 2–30 years). Six of the decision-makers were women and four were men. HCPs had been practicing on average for 11.4 years (range 5–25 years). Eight HCPs were women and two were men.

### Knowledge of the strategy

The level of knowledge regarding ATICA strategy was high among the majority of the health decision-makers and HCPs. The interviewees were able to describe both intervention components and the implementation process.

“The experience [with ATICA] was designed strategically and taking into account a number of factors: previous planning meetings, trainings, the type of message and how many times to send it” [Decision-maker 1].

“The majority of women came after the first message and if they didn’t they were sent another” [HCP 6].

### Characteristics of the intervention

#### Intervention source

The health decision-makers and HCPs positively assessed the fact that the ATICA study began as an initiative headed by CEDES (Centre for the Study of State and Society) in collaboration with Harvard University, institutions considered to have academic prestige and experience in mHealth research. Health decision-makers and HCPs highlighted that ATICA was designed and implemented collaboratively through articulation and consensus with provincial and national institutions (Argentinean National Cancer Institute, provincial Cancer Institute and PHC Direction). They also assessed positively their early engagement in ATICA implementation.

“[In workshops to implement ATICA], we listened and contributed ideas. We were there from the start” [Decision-maker 1].

“We never felt competition or like the idea was imposed. The provincial guidelines and ways of doing things were respected” [HCP 8].

Another positive aspect perceived by the majority of those interviewed was that ATICA began as a research study. In their opinion, this was a guarantee that scientific methods were applied to evaluate whether incorporation of mobile technology in PHC would work in the local context.

“The fact that ATICA begun as a research project was an advantage, because you can demonstrate that it works, there is an evaluation of results” [Decision-maker 5].

#### Design quality and Access to knowledge and information

Health decision-makers and HCPs mostly stated that ATICA strategy components worked in an articulated and complementary way, and that the training and information received were adequate. They also considered that the printed materials contained clear, concise information with graphics and drawings that favored an intuitive understanding and recall of the messages.

“The two options are complementary. If the patient doesn’t go to the health center after receiving the messages, it’s necessary for the community health worker to go see her” [HCP 6].

#### Adaptability

The majority of the interviewees agreed that adaptations to ATICA components were not necessary. It was unanimous that the content of the SMS was appropriate as it was clear, precise and in agreement with the legal norms regarding the communication of health information. They also considered adequate the frequency of the SMS.

“I wouldn’t change the content because the messages are respectful, subtle, and say “we need you to come” especially when the HPV result is positive” [HCP 10].

Regarding the e-mail/SMS sent to the CHWs to inform them of women without Pap triage after 60 days, the respondents agreed that sending WhatsApp messages would improve the communication and make it more dynamic. They also considered that this information should be sent to authorities with decision-making power (for example, the coordinator of the provincial CC prevention program, the PHC director and supervisors), so responsibility for assuring that women are triaged is shared with HCPs and CC prevention program authorities.

“Other members of the health team should also receive [the SMS], like the intermediate supervisors or the head of the CC prevention program, so CHWs do not bear all the responsibility [HCP 5].

#### Complexity and compatibility

The respondents predominantly evaluated ATICA as a low-complexity strategy and, at the same time, considered it compatible with the existing organization and way of functioning within health centers. In both cases, they agreed that the strategy was integrated into other processes that health services routinely perform (seek out, receive, and care for patients; inform medical results; input information regarding screening, triage, diagnosis and treatment in the national screening information system (SITAM); among others).

“ATICA doesn’t change or interfere with any process of the cervical cancer program” [Decision-maker 1].

“The strategy is compatible because it facilitates the community health care worker’s everyday work, and the professional continues the flowchart examining the positive self-collected tests” [HCP 1].

The interviewees also considered that the intervention was compatible with the forms of communication between health care services and women. In their opinion, this was evidenced by women’s high acceptance of the SMS as a channel of communication.

“Perfect as a form of communication. It absolutely favored the communication between the health center and the patient” [HCP 6].

There were other decision-makers and HCPs who perceived the strategy as complex due to the need to logistically coordinate the actions of different actors (CHWs, staff from the HPV test/cytology/histology laboratory, staff registering self-collection results in SITAM). They also perceived that the SMS system was complex, since to send SMS to HPV-positive and CHWs to visit women who had not had triage at 60 days it should be programmed to consider the screening/triage algorithm as well as the established timing between the steps.

#### Relative advantage

The interviewees mentioned several advantages –and no disadvantages– to the intervention in relation to the existing practices for communicating results to women with self-collected tests:

-Simplifying the process of delivering results increased adherence to timely triage.

“This lets the user know directly [about results availability] without being mediated by a doctor or a community health care worker who visits her. It makes the HPV-positive person attend more quickly” [HCP 1].

-Improving the health system’s communication with women, by sending information via a personalized, direct, opportune, and timely SMS.

“It is a marvelous intervention for facilitating communication. One hundred percent useful because the information mechanisms were greatly improved” [Decision-maker 4].

-Reducing the workload of CHWs in seeking out HPV + women without triage, especially those who require several visits to be reached. For the respondents, this meant a more efficient use of the human resources in the health system.

“Before this, the community health worker had to go four times to remind a woman to get a Papanicolaou. Now it’s four messages. Taking better advantage of human resources is extremely important” [HCP 8].

-Incorporating mobile phones in the provision of PHC services increased the willingness of staff at this level of care to adopt mobile technology in their work processes, with a benefit to the population.

“Now technology is an extension of the community health worker’s work and is somewhat more of a friend to primary health care” [Decision-maker 5].

#### Consideration of women’s needs

ATICA was mainly viewed as a strategy responding to women needs, by facilitating timely triage. Nevertheless, it was highlighted that the main barrier faced by some women to access triage was the lack of sample takers, and therefore the priority to meet women´s needs was to assure that in all health centers there was staff to take Paps. In this sense, they perceived that ATICA did not respond to a high-priority need.

“The priority for women is to not delay their appointment to get a Papanicolaou. ATICA was very well aimed at quick communication and information” [Decision-maker 8].

“Some women couldn’t get access [to a Pap test], and that is why it is necessary to guarantee greater coverage of professionals to take Pap tests” [HCP 5].

#### Relative priority

Both HCPs and decision-makers unanimously assigned a high priority to the incorporation of the ATICA strategy to ensure Pap triage and early diagnosis in women with positive self-collected tests. The strategy was seen as fundamental to complete the screening program goals in the target population.

“It is absolutely crucial in encouraging women to finish the path they started and get Pap triage” [Decision-maker 5].

#### Leadership engagement

Almost all interviewees considered that the provincial health authorities would commit to the programmatic incorporation of the strategy in the long term. In first place, the authorities have for years shown their commitment to CC prevention. Besides, the fact that authorities had formed part of the design and implementation of ATICA, in addition to the demonstrated effectiveness of the strategy, increased the possibility of commitment to the implementation.

“Yes, they will commit, we’ve been fighting for years against cervical cancer” [Decision-maker 5]

However, some decision-makers doubted the commitment of the provincial health authorities as they considered that, in public policy management, resolution of urgent matters prevail in detriment to other important issues like the low adherence to the CC care process. Additionally, their doubts had to do with the high rotation of health authorities. Thus, they considered that new authorities might not continue the lines of work developed by previous administrations.

#### External policies

In regard to the need for specific policies and regulations to extend ATICA at a provincial level, the opinions of health decision-makers were divided. On one hand, some considered it necessary, given that all programs have norms for functioning, target populations and procedures. Thus, to implement ATICA, guidelines regarding digital communication of the health system with the population would be necessary. This would facilitate ATICA’s uniform implementation in all health institutions.

In contrast, other decision-makers considered that the ATICA strategy could be carried out without additional regulations. They highlighted that the intervention was connected to an existing care protocol of CC screening, triage, diagnosis, and treatment.

“I don’t think [there is a need for] specific normative frameworks. [ATICA] has to do with a referral algorithm of positive self-collected tests that is already functioning” [Decision-maker 5].

#### Costs of ATICA implementation

The decision-makers identified three main items they believed necessary to fund for ATICA to function. The first is related to the operation and maintenance of the software for sending the SMS (MATYS).

“The program sending the text messages must have a cost, and logically sending the messages does too” [Decision-maker 2].

Second, they identified the provision of cell phones as a cost of ATICA strategy. Thus, for some decision-makers providing CHWs with cell phones would guarantee that they could do their job without having to take on costs. However, for other decision-makers, a cell phone should be provided to every health care facility. In this case, they considered that the institutional cell phone could be used for different health programs, and the funding would not fall exclusively to ATICA’s budget.

“Each CHW should be given a cell-phone and be provided with a mobile data package; some people might have problems using their own cell phone because that use has a cost” [Decision-maker 4].

“We need a cell phone per facility, not per community health worker. An institutional telephone at the health center for them to communicate with the health worker and the women” [Decision-maker 9].

A third cost had to do with the provision of free internet and WI-FI service to all health centers. For them, this was important to accessing data regarding screening, triage, diagnosis, and treatment through SITAM. However, on this issue, differences were found among decision-makers as some respondents considered that internet service was already widely available in health centers.

#### Maintenance

The interviewees perceived the programmatic incorporation of ATICA into the provincial CC prevention program as viable; they considered that the provincial health system had the organizational and technological conditions necessary to implement it, and that the usefulness of the ATICA strategy had been demonstrated. They also mentioned that the wide availability of cellular phones in the population would contribute to the programmatic scaling-up of the strategy.

“The implementation is very viable because we have a key actor that is the community health worker” [HCP 5].

“It should be incorporated because we saw that it is useful for the prevention of cervical cancer” [Decision-maker 2].

Several decision-makers and HCPs conditioned the viability of the programmatic incorporation of the intervention to the provision of cell phones to the health facilities and to CHWs, as well as to the possibility of connecting to internet and mobile data at no cost.

“With mobile devices, internet connection and mobile data in the health facilities… Otherwise, it won’t be very viable” [Decision-maker 3].

Regarding the strategy scaling-up, the interviewees agreed that it should be led by the provincial Ministry of Health (MoH), with ample involvement of PHC authorities and CHWs, the provincial CC prevention program, and the provincial Cancer Institute.

With regard to the elements necessary for guaranteeing ATICA’s programmatic functioning, the decision-makers agreed to assign importance to the political decision to prioritize CC prevention both by the provincial health authorities (MoH and hospital directors) and the Argentinean National Cancer Institute. They also highlighted the importance of the highest health authority, i.e., the provincial health minister, to establish ATICA as high priority line of work.

Additionally, the respondents agreed in highlighting the need to ensure human resources both for the HPV lab and for taking Pap tests at PHC centers, as well as the continued provision of HPV-tests and collectors.

“[To have ATICA strategy] as part of the cervical cancer prevention routine we need HPV kits always available, and the necessary reagents because otherwise the tests have to be suspended. And laboratory personnel so that the readings and results aren’t delayed” [HCP 4].

“We need to have gynecologists in all PHC centers, at least once a week” [Decision-maker 10].

## Discussion

Low levels of triage after a positive result have been identified as a major drawback of HPV self-collection. ATICA C-RCT resulted in a 15% increase in the percentage of women with triage Pap after the HPV result, showing that the multi-component mobile health intervention was effective in improving triage adherence. It also showed that the intervention was of high reach, adequately implemented, and highly accepted by HPV-positive women and adopted by CHWS [23]. However, to fully capture the likelihood of the intervention being implemented and sustained over time, it was essential to evaluate the perceptions of health decision-makers and HCPs regarding implementation and scaling-up of ATICA strategy. To our knowledge, this is the first analysis of the perspective of health decision-makers and HCPs regarding a multicomponent mHealth intervention to increase adherence to follow-up among women with HPV self-collected tests. The study design combined the application of CFIR [25] and RE-AIM [29], two conceptual frameworks from implementation science appropriate for evaluating implementation and sustainability of health interventions. Results showed that both HCPs and decision-makers had a positive assessment of the intervention through most included constructs. However, some potential barriers were identified in complexity, leadership engagement, external policies, economic cost and maintenance constructs. Thus, stakeholders conditioned the strategy’s sustainability to the political commitment of national and provincial health authorities to prioritize CC prevention, and to the establishment of the ATICA strategy as a programmatic line of work by health authorities. They also highlighted the need to ensure, above all, the availability of staff to take the Pap tests and carry out the HPV-lab work, and to guarantee a constant provision of HPV-test.

The evidence shows that the legitimacy of the origin of an intervention is associated with success in its implementation [36]. Key ideas for improving health care provision that come from outside the organization and that are then effectively tailored to the organization have been highly related to successful implementation [37]. In our study, stakeholders considered that ATICA strategy was proposed by prestigious external institutions and then designed and implemented collaboratively through consensus with local institutions and health providers. In addition, interviewees highly valued the fact that local stakeholders were involved in the design and implementation of the intervention since its early stages. Respondents also pointed out the importance of ATICA as a research study that would provide data on the strategy effectiveness. Integrating research activities into existing health systems with involvement of local stakeholders, as done in ATICA study, has been identified in the literature as an important factor for successful incorporation of evidence-based interventions [38, 39]. The perception of collaborative work and early involvement might also be factors that explain why respondents did not perceive the need for substantial adaptations of the intervention.

Our study found that for decision-makers and HCPs the implementation of ATICA would offer important relative advantages in relation to the current programmatic situation regarding delivery of results. These findings are important because stakeholder’s acknowledgement of the relative advantages of an intervention constitute a *sine qua non* for implementation/adoption [40]. A study carried out in Canada [41] showed that a main factor facilitating adoption on the part of clinicians of a mobile application for monitoring heart patients was the perception that the application had several advantages over other telemonitoring systems. In our study, no disadvantages were identified, probably because the existing procedure for result delivery by which HPV-tested women are instructed to go to the health center within 30 days to pick up results has been shown to be highly deficient [42–44]. The intervention was perceived as implying a more efficient use of PHC resources, with a reduction in CHW workload and an improvement in the communication of self-collection results. The fact that in the ATICA strategy CHWs only visit positive women who have not responded to the SMS messages was perceived as an advantage over the existing situation as at present, very often CHWs are asked to visit all non-adherent positive women, which results in the incorporation of a high number of unplanned visits into their daily tasks.

The perception of the relative advantage of the intervention over the existing situation must be shared by all involved actors to assure effective implementation [40]. Undoubtedly, a key stakeholder in ATICA strategy scaling-up are the CHWs, as implementation of both intervention components is highly dependent on them explaining the intervention during the offer of HPV self-collection, and the visit to positive women who have not triaged at 60 days to be included as part of their tasks. Acceptability of interventions by CHWs is correlated with potential work overburden that those might impose on their daily tasks [45]. Although our study did not interview CHWs, previously reported results showed that 87% of CHWs accepted the incorporation of the ATICA strategy into their daily activities and 91% visited HPV + women who did not respond to the SMS [23]. It is therefore possible to assume that interviewees’ perception of the intervention as advantageous for CHWs is also shared by CHWs.

The complexity of an intervention and its compatibility with existing practices are concepts related to factors that can promote or hinder implementation [46]. Evidence has shown that when there is a good perceived fit between e-health systems and workflows, and when systems positively influence workplace efficiency, their use is facilitated [47]. Our results showed that the intervention was perceived both by health decision-makers and HCPs as highly compatible with PHC existing organization and ways of functioning. They also agreed that the ATICA strategy could be easily integrated into processes that health services regularly carry out. Complexity is also increased by the process length, i.e. the number of sequential subprocesses and actors involved in health service provision [48]. This process complexity was signaled by some of the interviewees by highlighting that the SMS is a simple tool for communicating with women and CHWs which is integrated into a multi-step screening process. Thus, our results suggest that, for successful integration of the ATICA strategy into CC screening programs, coordination of actors, institutions and activities involved in the screening continuum should be assured.

Our study also identified some potential obstacles related to the costs of implementing the ATICA strategy, as mHealth interventions require ongoing technological maintenance and support [49]. In the ATICA study, the cost of operating MATYS, the SMS system, was met by the research study, but in the strategy scaling-up the cost should be integrated into provincial/national MoH budgets. This could represent an obstacle for assuring scaling-up of the intervention. Eze et al. [50] have shown that educating health decision-makers regarding the long-term benefits of mHealth tools might favor long-term financial support by increasing health authorities understanding of clear return of investment for mHealth technologies and reasoning out the potential for future projects. In the ATICA study, both national and provincial health authorities were involved in the intervention design, implementation, and evaluation. Along the project, several meetings and workshops were carried out regarding ATICA implementation, which were also an opportunity to discuss with them about the role of mHealth interventions in reducing unequal access to cervical cancer prevention and how the multi-component intervention could be latter adapted for increasing continuity in the care continuum of other tumors or health conditions. Therefore, following Eze et al. [50] we might expect that these activities will increase shared values about using mHealth technology, with a positive impact on the long-term financial support.

Interviewees in our study raised the issue of up to what extent an mHealth strategy based on PHC should rely on CHWs personal cell phones and access to internet. This is certainly a point to be considered when designing mHealth strategies, especially in settings with unequal access to internet across the health system. As suggested by some interviewees, a solution might be to have institutional cell phones at health centers to be shared by CHWs and other health services. However, the high level of coordination needed for this might be difficult to achieve in LMIC health systems. As a result, CHWs might end up with the additional burden of having to assure access to cellphones whenever they need them. Providing professional phones might be an alternative solution, however this might create new concerns for CHWs as protecting the phones from loss, theft or damage can be challenging [51]. Feasibility of assuring access to mobile data by CHWs should be evaluated.

In CFIR, inner setting related constructs allows to understand how factors related to the organizational context might associate with implementation and scaling-up of an evidence-based intervention. Maintenance is a RE-AIM dimension that refers to the degree of institutionalization of an intervention, to the point that it begins to form part of the routine practices of an institution or organization [29]. Used together, RE-AIM and CFIR allow elucidating relationships between factors which potentially could lead to optimal post-trial maintenance outcomes [52]. In our study, interviewees’ perceptions suggested a likely high level of post-trial adoption of the intervention, as they assigned a high relative priority to the intervention implementation and perceived a high potential commitment of health authorities with its scaling-up. Nonetheless, interviewees also mentioned the high turnover of policy makers as a note of concern regarding future health authorities’ commitment with the scalability of the strategy. In the case of the ATICA strategy, policy-maker high turnover might have a negative impact in its scaling-up, as establishing the strategy as a programmatic activity will be highly dependent on decisions that have to be taken at high level health authorities. An example of this is the inclusion of the visit of the CHWs to women who do not have Pap triage at 60 days as part of the routine PHC activities, or incorporating and funding the SMS sending system as a component of the national screening information system (SITAM) so SMS can be sent. Since completion of ATICA C-RCT in 2019, national highest health authorities in charge of cancer control changed several times. Although collaborative work to plan scaling-up activities was initiated [23] each change of health authority has slowed down the actual incorporation of the strategy as a programmatic routine activity. Thus, our study underscores the need to produce evidence about implementation of effective strategies to increase maintenance of an evidence-based intervention in contexts of high turnover of health authorities.

In our study, respondents valuated the ATICA strategy as highly effective in increasing triage of HPV + women and facilitating access to an early diagnosis; this assessment coincides with the results of the C-RCT [23]. This is a key result, as the perception of effectiveness of the intervention is an influential factor of the intervention’s sustainability [53, 54]. Research on the use of mobile phones for sending SMS, voice and video messages among patients and HCPs [55] showed that the perception of effectiveness of the intervention on the part of patients, decision-makers and HCPs facilitated its sustainability in different health systems (urban and rural environments in Canada, Kenya and Uganda). A systematic review that evaluated the use of tele-homecare for the management of chronic diseases showed that uncertainty regarding the clinical effectiveness of this type of interventions by key actors was a barrier to its sustainability [56].

Regarding the extent to which the intervention responded to women’s needs, respondents recognized the importance of the ATICA strategy to facilitate access to timely triage. Nonetheless, they also signaled that women’s first priority was the health system to guarantee human resources for taking triage Paps and processing HPV-tests, and the constant provision of HPV-tests and related supplies. These findings are consistent with the literature, which shows that the provision of supplies and human resources are key factors that influence the sustainability of any health intervention [53]. The availability of supplies and human resources for taking and reading Pap tests has historically been highlighted as one of the primary barriers to CC screening [57–59]. A study carried out in Malawi, that used CFIR to evaluate the implementation of a screening and treatment program to prevent CC found that resource availability (the lack of human resources as well as supply shortages) negatively affected the CC screening program implementation [60]. The introduction of HPV-testing has served as a tool for improving the organizational capacity of CC prevention programs [8, 61]. A study carried out in Argentina showed that after the introduction of the HPV-test, problems related to cytology technical staff and supplies for reading Pap tests were reduced [62]. Additionally, introduction of HPV self-collection reduced problems related to the lack of screening sample takers [62]. Nevertheless, variations in the dollar exchange rate and national policies restricting importation during certain periods disrupted the purchase of HPV reagents, which led to interruptions in the availability of the test. Thus, the perception of the stakeholders regarding the need to guarantee essential resources for the screening system to work is a recognition that ATICA strategy should be implemented in the context of a CC prevention program that works adequately and has the necessary resources. Guarantying a permanent and uninterrupted supply of HPV-testing should be a priority.

A limitation of the study is that it evaluated the perspective of health decision-makers and HCPs in a specific setting, which limits the generalization of the results. In this sense, future research is needed to adjust the ATICA strategy to other health systems, local contexts and population needs. Also, our study did not interview national health authorities involved in cervical cancer prevention, so perception of this key actor might differ from provincial decision-makers. However, the research team is working with authorities of the Argentinean National Cancer Institute on scaling-up of the ATICA strategy, suggesting that the positive perception of provincial policy makers identified in our study also applies to national health authorities.

## Conclusion

Our study showed a highly positive assessment on the part of health decision-makers and HCPs regarding a multi-component mobile health intervention that is effective to improving triage adherence among women with self-collected tests offered by CHWs. The intervention could be adapted to increase compliance of other important steps of the cervical prevention process, such as colposcopy/biopsy and treatment. In addition, it might also be adapted for implementation in other cancer prevention programs, such as colon and breast cancer control programs.

The results obtained allow for a better understanding of factors that facilitate implementation and scaling-up of mHealth interventions rooted in PHC, that are aimed at strengthening the CC screening/diagnosis/treatment care continuum. These results have important implications for middle and low-income countries implementing or considering implementing HPV self-collection with mHealth interventions to ensure high adherence to follow-up and make progress toward the WHO targets to eliminate cervical cancer.

## Supplementary Information

**Additional file 1.**

**Additional file 2.**

**Additional file 3.**

## Data Availability

The datasets generated during the current study are not publicly available in order to protect respondent confidentiality, but are available from the. corresponding author on reasonable request.

## Abbreviations

ATICA: Application of Communication and Information Technologies to Self-Collection
CC: Cervical cancer
CEDES: Centre for the Study of State and Society
CEMIC: Dr. Norberto Quirno Research Ethics Committee
CFIR: Consolidated Framework for Implementation Research
CHWs: Community health workers
C-CRT: Cluster-randomized trial
HCPs: Health-care providers
HPV: Human papillomavirus
HPV+: Human papillomavirus positive
MATYS: Software for sending the mobile phone text messages
MoH: Ministry of Health
Pap: Papanicolaou
PHC: Primary health care
RE-AIM: Reach Effectiveness Adoption Implementation Maintenance
SITAM: National Screening Information System
SMS: Mobile phone text messages
WHO: World Health Organization
