# Supplementary material for: Implementation and scaling-up of an effective mHealth intervention to increase adherence to triage of HPV-positive women (ATICA study): perceptions of health decision-makers and health-care providers
Source: BMC Health Serv Res. 2023 Jan 18;23:47. doi: 10.1186/s12913-023-09022-5 (PMC9847147; doi:10.1186/s12913-023-09022-5)

| **Additional file 1: ATICA study, Interview guides** | | |
| --- | --- | --- |
| **Health care providers** |  | **Health decision-makers** |
| - Profession/speciality: - Institution where you work: - Sphere (provincial; health region; municipal): - Position held: - Length of service: - Description of your daily tasks: In your professional activity, which one is related to CC? Do you have any other professional experience (clinical practice, program management, or similar) related to cervical cancer (CC) prevention in particular? - Role during ATICA project (in 2019): |  | - Profession/speciality: - Unit/Institution where you work: - Sphere (provincial; health region; municipal): - Position held: - Length of service: - Description of your role/tasks in this position: - What professional experience (clinical practice, program management, or similar) do you have in relation to cervical cancer (CC) in particular? - Role during ATICA project (during 2019): |
| First of all, I will ask you about cervical cancer prevention  **Q1.** What **CC prevention measures are being developed in the province**?  **Q2**. With regard to the care of HPV+ women, i.e. triage, diagnosis and treatment of women who need it, what actions are being taken? What factors **facilitate** the adoption of these measures and what factors **hinder** them?  **Q3**. And specifically, when faced with a case of HPV-positive self-collection (SC+), what measures/actions are taken to ensure that SC+ women carry out the triage Pap in the province? In your professional practice do you have any particular strategy to solve this issue? (Aims to identify the micro strategies that are carried out in PHC to perform the triage Pap). |  | First of all, I will ask you about cervical cancer prevention.  **Q1.** What **CC prevention measures** are being developed in your sphere of action (province; department; municipality; district)?  **Q2.** With regard to the care of HPV+ women, i.e. triage, diagnosis and treatment of women who need it, what actions are being taken? What factors **facilitate** the adoption of these measures and what factors **hinder** them?  **Q3.** And specifically, when faced with a case of positive self-collection, what measures/actions are taken to ensure that SC+ women carry out the triage Pap in the province? (Aims to identify the micro strategies that are carried out in the PHC to perform the triage Pap). |
| **Q4.** Taking into account the care of women with SC+, the performance of the Triage Pap: Do you consider that **changes are necessary**? In what aspects? Why? (Take into account the practice referred to in question Q2) |  | **Q4.** Taking into account how adherence to Pap triage for women with SC+ is handled, do you consider that **changes are necessary**? In what aspects? Why? |
| Recalling, ATICA project was aimed to send SMS to women with a SC+ to inform them that their result was available at the health center and to encourage them to go to there and, in cases where women did not have a Pap within 60 days, Health Agents received an SMS with contact information.  **[Show Card with the intervention]** -Figure 1-  **Q5.** In general terms, what is **your opinion** about this intervention? Do you think the intervention can be useful for the care of women with SC+? In what way? |  | Recalling, ATICA project was aimed to send SMS to women with a positive self-collection (AT+) to inform them that their result was available at the health center and to encourage them to go to the health center and, in cases where women did not have a Pap within 60 days, Health Agents received an SMS with contact information.  **[Show Card with the intervention]** -Figure 1-  **Q5.** In general terms, what is **your opinion** about this SMS intervention for women with SC+? Do you think that sending an SMS can be useful for the care of women with SC+? In what way? |
| **Q6.** How did you find out about/know about ATICA Project?  **a)** Did you have the opportunity to attend an information activity? Did you receive information material (leaflet, note from PHC)?  **b)** How do you evaluate the information received? |  | **Q6.** How did you find out about/know about ATICA Project?  **a)** Did you have the opportunity to attend an information activity (meeting with the research team, training, etc.)? Did you receive information material (note, leaflet)?  **b)** How do you evaluate the information received? |
| **Q7.** Thinking about the population you serve, what do you think are the **priority needs** related to the care process for women with SC+?  **a**) Taking this into account, to what extent can ATICA strategy respond to these needs? |  | **Q7.** Thinking about the characteristics of the population in your region/province, what do you think are the priority needs related to the care process for women with SC+?  **a)** Taking this into account, to what extent can ATICA strategy respond to these needs? |
| **Q8.** Considering the strategies being developed to ensure triage Pap smears for women with SC+, what **advantages** do you see in this **text messaging** intervention? And what, in your opinion, have been the **disadvantages**?  **Q9.** How high a priority would be the incorporation of ATICA strategy to ensure the accomplishment of the triage Pap for women with SC+? |  | **Q8.** Considering the strategies being developed to ensure triage Pap smears for women with SC+, what **advantages** do you see in this **text messaging** intervention? And what, in your opinion, have been the **disadvantages**?  **Q9.** How high a priority would be the incorporation of ATICA strategy to ensure the accomplishment of the triage Pap for women with SC+? |
| **Q10.** Do you consider ATICA strategy is **compatible** with your health center's organizational and operational methods, or does it contradict them? Why?  Is sending SMS as a strategy compatible with the forms of communication between health services and women, or does it contradict them? Why? |  | **Q10.** Do you consider ATICA strategy is **compatible** with your health center's organizational and operational methods, or does it contradict them? Why?  Is sending SMS as a strategy compatible with the forms of communication between health services and women, or does it contradict them? Why? |
| **Q11.** How do you evaluate the growing use of cell phones for patient-physician communication?  **Q12.** Do you know of other experiences with the use of text messages in the health care sector?  **a)** [If you do know] What is your opinion about these experiences? Have any of them been carried out in Jujuy?  **b)** Can these experiences be a stimulus to implement ATICA strategy?  [Note: here we want to know the influence that other implementation experiences may have in relation to other health issues (diabetes, for example) and whether or not they may be a factor that "motivates" the implementation of ATICA strategy and why]. |  | **Q11.** Do you know of other experiences with the use of text messages in the health care sector?  **a)** [If you do know] What is your opinion about these experiences? Have any of them been carried out in Jujuy?  **b)** How do you evaluate the growing use of cell phones for patient-physician communication?  [Note: here we want to know the influence that other implementation experiences may have in relation to other health issues (diabetes, for example) and whether or not they may be a factor that "motivates" the implementation of ATICA strategy and why]. |
| **Q13.** The fact that it started as an initiative led by CEDES (research institute), in collaboration with Harvard University, the National Cancer Institute and the Ministry of Health of Jujuy, to what extent do you consider it an advantage? And what could be the disadvantages? And the fact that it started as a research project, do you consider it an advantage or a disadvantage?  **Q14.** In your opinion, this initiative led by CEDES (research institute), in collaboration with Harvard University, the National Cancer Institute and the Ministry of Health of Jujuy, **to what extent does it respond to a need of the health services** to communicate with women? Why?  **Q15.** If ATICA were to be extended to the whole province, which institutions and actors should be involved in its implementation?  [Note: an intervention that was conceived by a scientific body is perceived as legitimate. It also inquiries about who should be involved for it to have legitimacy]. |  | **Q12.** In your opinion, sending SMS to women with SC+ to promote the Pap triage procedure, **to what extend does it respond** **to a** **need of the health services** to communicate with women? Why?  **Q13.** The fact that it started as an initiative led by CEDES (research institute), in collaboration with Harvard University, the National Cancer Institute and the Ministry of Health of Jujuy, to what extent do you consider it an advantage? And what could be the disadvantages? And the fact that it started as a research project, do you consider it an advantage or a disadvantage?  **a)** In your opinion, if ATICA were to be extended to the whole province, which institutions and actors should be involved in its implementation?  [Note: an intervention that was conceived by a scientific body is perceived as legitimate. It also inquiries about who should be involved for it to have legitimacy]. |
| **[Show Card with the intervention/ SMS content]** -Figures 1 and 2-  **Q16.** Thinking about the two components (sending SMS and visiting women without triage by the HWs do you think one might work better than the other?  **Q17.** Taking into account what has been done in the framework of ATICA strategy and considering the population you serve; what **adaptations or changes would you suggest** to this strategy?  **a)** What **adaptations** should be applied to the SMS: frequency of sending SMS, quantity of SMS sent, SMS content?  **b)** Regarding the role of health workers in the intervention (Offering SC and visiting women who did not perform triage, how?  **c)** For communication with HWs who are to visit women without triage, should other means be used in addition to/instead of SMS and mail (e.g., WhatsApp)?  **d)** Do you consider messages / mails should be sent to **other actors**? Who? |  | **[Show Card with the intervention/ SMS content]** -Figures 1 and 2-  **Q14.** Thinking about the two components (SMS sending and non-adherent cases retrieval by HWs) do you think one might work better than the other?  **Q15.** Taking into account what has been done in the framework of ATICA strategy and considering the population of the province, what **adaptations or changes would you suggest** to this strategy?  **a)** What adaptations should be applied to the SMS: frequency of sending SMS, quantity of SMS sent, SMS content?  **b)** Regarding the role of health workers in the intervention (Offering SC and visiting women who did not perform triage, how?  **c)** For communication with HWs who are to visit women without triage, should other means be used in addition to/instead of SMS and mail (e.g., WhatsApp)?  **d)** Do you consider messages / mails should be sent to **other actors**? Who? |
| **Q18.** Thinking about ATICA strategy in terms of its components and the actors involved, would you say that it is a **simple** or **complex** intervention? Which aspects are simple/complex? Why? |  | **Q16.** Thinking about ATICA strategy in terms of its components and the actors involved, would you say that it is a **simple** or **complex** intervention? Which aspects are simple/complex? Why? |
|  |  | **Q17.** In terms of budget, what would need to be financed in order to implement ATICA throughout the province? |
|  |  | **Q18.**In your opinion, what are the costs of implementing a text message strategy (usage of human resources, necessary inputs, cost of the SMS sending system)? |
| **Q19.** To what extent do you think ATICA strategy can be sustained over time? Do you think it is feasible to implement it throughout the province? What would need to be ensured for it to work as part of the routine for CC prevention (screening, diagnosis, follow-up and treatment)? |  | **Q19.** To what extent do you think ATICA strategy can be sustained over time? Do you think it is feasible to implement it throughout the province? And at national level?   - What would need to be ensured for it to work as part of the routine for CC prevention (screening, diagnosis, follow-up and treatment)? - From the role you play in the health system, what aspects do you think could contribute for ATICA strategy to last over time? |
| **Q20.** In your opinion, which institution should promote/lead the programmatic implementation of ATICA strategy for it to be accepted and incorporated by professionals throughout the province? (Ministry/Secretariat of Health; Professional Associations; Civil Society Organizations; International Organizations). |  | **Q20.** In your opinion, which institution should promote/lead the programmatic implementation of ATICA strategy for it to be accepted and incorporated by professionals throughout the province? (Ministry/Secretariat of Health; Professional Associations; Civil Society Organizations; International Organizations). What agreements or strategic alliances should be established?  **Q21.** Do you consider that any specific rule or regulation may be necessary? |
| **Q21.**Do you think that the local health system authorities will be committed to the adoption of ATICA strategy in the long term? Why? What elements make you think that this would be the case? |  | **Q22.** Do you think that the local health system authorities will be committed to the adoption of ATICA strategy in the long term? Why? What elements make you think that this would be the case? |
| **Q22.** Would you like to add anything else? |  | **Q23.** Would you like to add anything else? |

***
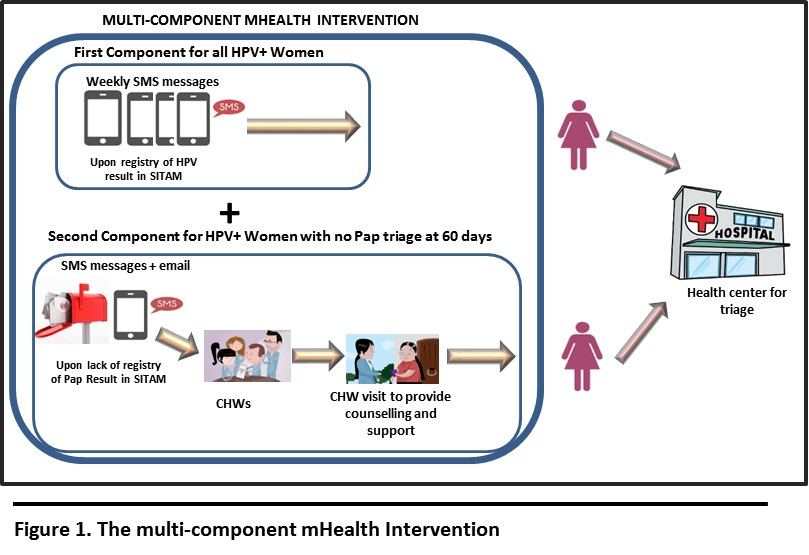
***

**Figure 2. Messages for women who test HPV negative or positive**


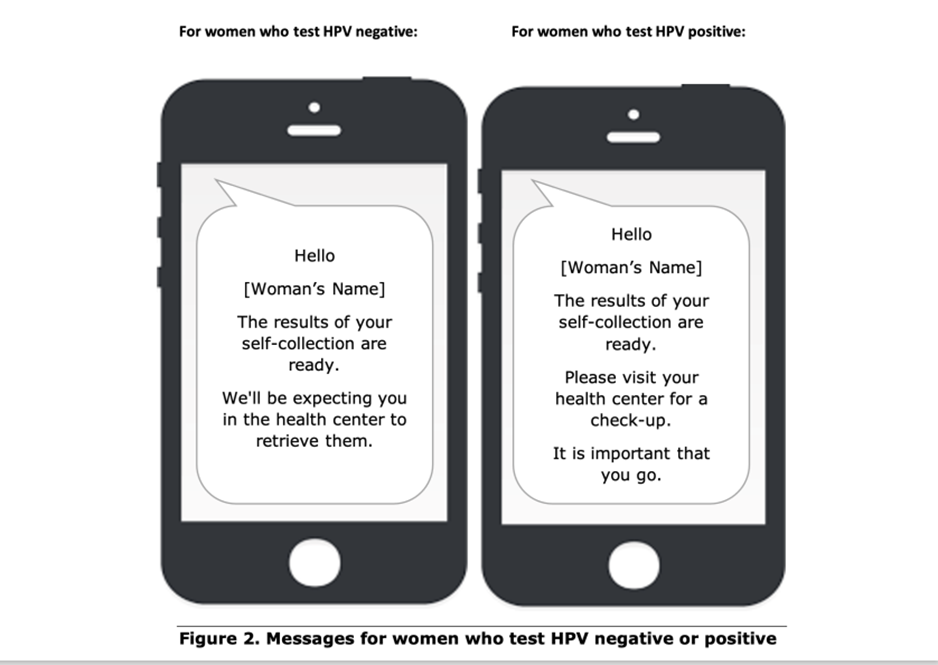

Supplement: Supplementary file 1 — Additional file 1. [file 12913_2023_9022_MOESM1_ESM.docx]
