# Supplementary material for: Implementation and scaling-up of an effective mHealth intervention to increase adherence to triage of HPV-positive women (ATICA study): perceptions of health decision-makers and health-care providers
Source: BMC Health Serv Res. 2023 Jan 18;23:47. doi: 10.1186/s12913-023-09022-5 (PMC9847147; doi:10.1186/s12913-023-09022-5)
Supplement: Supplementary file 2 — Additional file 2. [file 12913_2023_9022_MOESM2_ESM.docx]

**Additional file 2: Consolidated criteria for reporting qualitative studies (COREQ): 32-item checklist**

No Item Guide questions/description

**Domain 1: Research team and reflexivity**

Personal Characteristics

1. Interviewer/facilitator: Which author/s conducted the interview or focus group?

First Author

2. Credentials: What were the researcher’s credentials?

PhD

3. Occupation: What was their occupation at the time of the study?

Independent Researcher

4. Gender: Was the researcher male or female?

Female

5. Experience and training: What experience or training did the researcher have?

Researcher in social sciences applied to the field of health.

Relationship with participants

6. Relationship established: Was a relationship established prior to study commencement?

No.

7. Participant knowledge of the interviewer: What did the participants know about the researcher?

The participants were informed that the researcher was doing interviews as part of the ATICA study.

8. Interviewer characteristics: What characteristics were reported about the interviewer/facilitator? e.g. Bias, assumptions, reasons and interests in the research topic

We reported that the interviewer was a researcher trained in qualitative research.

**Domain 2: study design**

Theoretical framework

9. Methodological orientation and Theory: What methodological orientation was stated to underpin the study?

Thematic analysis.

Participant selection

10. Sampling How were participants selected?

The sample was purposive.

11. Method of approach: How were participants approached?

The participants were approached through mail or WhatsApp to invite them to participate in a virtual interview, which was carried out through a virtual platform.

12. Sample size: How many participants were in the study?

20 participants: 10 health decision-makers and 10 health care providers.

13. Non-participation: How many people refused to participate or dropped out? Reasons?

A health decision-maker declined the interview due to lack of time due to work responsibilities during the Covid 19 pandemic.

Setting

14. Setting of data collection: Where was the data collected?

The data was collected through a virtual platform and most of the interviewees were at their workplaces.

15. Presence of non-participants: Was anyone else present besides the participants and researchers?

No other person was present during the interviews besides the respondent and the researcher.

16. Description of sample: What are the important characteristics of the sample?

The most important characteristic of the sample was the role played by the interviewees during the ATICA Project (health decision-makers and health care providers).

Data collection

17. Interview guide:

Were questions, prompts, guides provided by the authors? Was it pilot tested?

The interviews were conducted using a guideline with questions prepared considering the relevant dimensions and constructs of the CFIR and RE-IAM (Table 1). To streamline the interview, participants were shown three cards that graphed the mHealth multicomponent of the intervention, and the contents of the SMS sent to the women when the result of the self-collection was positive or negative. The guideline was not pilot tested.

18. Repeat interviews: Were repeat interviews carried out? If yes, how many?

No interview was repeated.

19. Audio/visual recording: Did the research use audio or visual recording to collect the data?

Yes, the research used audio recording.

20. Field notes: Were field notes made during and/or after the interview or focus group?

The research did not make fields notes during the interviews.

21. Duration: What was the duration of the interviews or focus group?

The duration of the interviews was one hour on average.

22. Data saturation: Was data saturation discussed?

Yes.

23. Transcripts returned: Were transcripts returned to participants for comment and/or correction?

No.

**Domain 3: analysis and findings**

Data analysis

24. Number of data coders: How many data coders coded the data?

Transcripts were coded independently by two researchers to later compare, debate, and resolve the inconsistencies with the other.

25. Description of the coding tree: Did authors provide a description of the coding tree?

Yes. Table 1 of the manuscript presents the domains, constructs and theoretical dimensions that guided the coding.

26. Derivation of themes: Were themes identified in advance or derived from the data?

The themes were identified in advance in the guidelines prepared considering the dimensions and the relevant theoretical constructs.

27. Software: What software, if applicable, was used to manage the data?

The software used was ATLAS.ti (version 7.5.4; ATLAS.ti Scientific Software Development GmbH, Berlin).

28. Participant checking: Did participants provide feedback on the findings? No.

Reporting

29. Quotations presented: Were participant quotations presented to illustrate the themes / findings? Was each quotation identified?

Yes, quotes from the participants are presented to illustrate the themes. The citations identify the type and number of the respondent (e.g., decision-maker 1; health care provider 8 -HCP 8-).

30. Data and findings consistent: Was there consistency between the data presented and the findings?

Yes.

31. Clarity of major themes: Were major themes clearly presented in the findings?

Yes.

32. Clarity of minor themes: Is there a description of diverse cases or discussion of minor themes?

Yes.
