# Supplementary material for: Implementation and scaling-up of an effective mHealth intervention to increase adherence to triage of HPV-positive women (ATICA study): perceptions of health decision-makers and health-care providers
Source: BMC Health Serv Res. 2023 Jan 18;23:47. doi: 10.1186/s12913-023-09022-5 (PMC9847147; doi:10.1186/s12913-023-09022-5)
Supplement: Supplementary file 3 — Additional file 3. [file 12913_2023_9022_MOESM3_ESM.docx]

**Additional file 3: CFIR and RE-AIM Domains, constructs, themes and results**

|  | | **Results** | |
| --- | --- | --- | --- |
| **CFIR Domains/ Construct** | | **Themes** | **Quotes** |
| **I. Intervention characteristics** | |  | |
| Intervention Source | | ATICA led by external prestigious institutions (+) | “The advantage is that there are highly prestigious institutions involved.” |
|  |  | ATICA designed and implemented collaboratively with local institutions involved at early stages of the project (+) | “We listened and contributed ideas. We were there from the start.” |
|  |  | ATICA designed as a research study (+) | “The fact that ATICA begun as a research project was an advantage.” |
| Relative advantage | | Simplification of results delivery;  increased adherence to timely triage (+) | “This lets the user know directly [about results availability]. It brings the HPV-positive person in more quickly.” |
|  | | Improvement of the health system’s communication with women (+) | “It is a marvelous intervention for facilitating communication.” |
|  | | Reduction in CHW workload (+) | “Before this, the community health worker had to go four times to remind a woman to get a Papanicolaou. Now it’s four messages.” |
|  | | Increased willingness of PHC staff to adopt mobile technology in their work (+) | “Now technology is an extension of the community health worker’s work.” |
| Adaptability | | No need to adapt the multicomponent strategy (+)  SMS should also be sent to other health authorities (+) | “I wouldn’t change the content because the messages are respectful, subtle…”  “Other members of the health team should also receive [the SMS], so CHWs do not bear all the responsibility.” |
| Complexity | | Low-complexity due to ATICA integration into health centers routine activities (+) | “ATICA doesn’t change or interfere with any process of the cervical cancer program.” |
|  | | High complexity linked to the need to logistically coordinate different actors (-) | “It is very complex as it involves the work of different health institutions and care providers.” |
|  | | High complexity linked to the need to articulate the SMS sending system, algorithms, and later steps of the continuum of care (-) | “[ATICA] needs a logistic to send messages depending on the HPV result, and then the organization of follow-up in proper times.” |
| Design Quality | | ATICA components well designed, they were articulated and complementary (+)  Clear printed materials about the intervention (+) | “The two components are well designed, they are complementary.”  “Brochures had clear and concise information, with drawings and graphics and few written contents.” |
| Perceived costs and necessary resources | | The operation and maintenance of the software for sending the SMS (MATYS). (*) | “The program sending the text messages must have a cost, and logically sending the messages does too.” |
|  |  | Provision of cell phones to CHWs or to health care facilities (*) | “Each CHW should be given a cell-phone and be provided with a mobile data package.” |
|  |  |  | “We need a cell phone per facility, not per CHW.” |
| **II. Outer Setting** | |  | |
| Patient needs | | ATICA strategy facilitates timely triage (+) | “The priority for women is to not delay their appointment to get a Pap. ATICA was very well aimed at quick communication and information.” |
|  |  | Main women need is an increased access to timely triage (-) | “It is necessary to guarantee greater coverage of professionals to take Pap tests.” |
| External policies and incentives | | Regulations are necessary for ATICA scaling-up (-) | “We need regulations stating whom the SMS will be sent to, what to do once results are available at the health center and how they will be delivered to women.” |
|  |  | No need of regulations for ATICA scaling-up (+) | “[ATICA] has to do with a referral algorithm of positive self-collected tests that is already functioning.” |
| **III. Inner Setting** | |  | |
| Access to knowledge and information | | Adequate training and information (+) | “All staff was trained. […] They gave us a bag containing a folder with all the information about the project, it was very good.”  “Brochures were very good and very informative; they were very helpful to inform the community.” |
| Implementation climate | Compatibility | ATICA compatible with the existing health system organization (+) | “ATICA doesn’t change or interfere with any process of the cervical cancer program.” |
|  |  | ATICA compatible with the forms of communication between health centers and women (+) | “It absolutely favored the communication between the health center and the patient.” |
|  | Relative priority | High priority incorporation of the ATICA strategy (+) | “It is absolutely crucial in encouraging the women to finish the path they started and get Pap triage.” |
| Readiness for Implementation | Leadership engagement | Commitment of health authorities with ATICA scaling-up (+) | “Yes, they will commit, we’ve been fighting for years against cervical cancer.” |
|  |  | Doubts regarding commitment of health authorities with ATICA scaling-up due to high turnover (-) | “It is difficult to know if they will commit because they are changing all the time.” |
| **IV. Characteristics of Individuals** | |  | |
| Knowledge and beliefs about the intervention. | | Knowledge regarding the ATICA strategy was high (+) | “[ATICA involved]: previous planning meetings, trainings, the type of message and how many times to send it.” |
| **RE-AIM Dimension** | |  | |
| Maintenance | | Viable Incorporation of ATICA into the provincial CC prevention (+) | “The implementation is very viable because we have a key actor that is the community health worker.” |
|  |  | ATICA as a tool for improving CC prevention (+) | “It is useful for the prevention of cervical cancer.” |
|  |  |  |  |
|  |  | *Scaling-up viability linked to:*  Political commitment of health authorities to prioritize CC prevention and establish ATICA as programmatic line of work, with ample involvement of PHC (*) | “For ATICA sustainability, cervical cancer prevention should be a prioritized by health authorities at highest level.”  “For ATICA to work it has to be led by PHC.” |
|  |  | Provision of mobile technology and internet (*) | “With mobile devices, internet connection and mobile data in the health facilities… Otherwise, it won’t be very viable.” |
|  |  | Ensuring human resources, and provision of HPV-test (*) | “We need to have gynecologists in all PHC centers, at least once a week.” |
|  |  |  | “[…] We need HPV kits always available… and laboratory personnel so… results aren’t delayed.” |

(+) positive perception; (-) negative perception; (*) perception of costs/resources needed to scale-up and maintain the ATICA strategy
